# Plant effects on microbiome composition are constrained by environmental conditions in a successional grassland

**DOI:** 10.1186/s40793-024-00550-z

**Published:** 2024-01-24

**Authors:** Lenka Mészárošová, Eliška Kuťáková, Petr Kohout, Zuzana Münzbergová, Petr Baldrian

**Affiliations:** 1 Institute of Microbiology of the CAS, v. v. i., Vídeňská 1083, Prague 4, 142 20 Czech Republic; 2 University of Chemistry and Technology, Technická 5, Praha 6, 166 28 Czech Republic; 3 Institute of Botany of the CAS, v. v. i., Zámek 1, Průhonice, 252 43 Czech Republic; 4 Department of Botany, Faculty of Science, Charles University in Prague, Benátská 2, Prague 2, 128 01 Czech Republic; 5 Department of Forest Ecology and Management, Swedish University of Agricultural Sciences, Skogsmarksgränd 17, Umeå, 901 83 Sweden

## Abstract

**Background:**

Below-ground microbes mediate key ecosystem processes and play a vital role in plant nutrition and health. Understanding the composition of the belowground microbiome is therefore important for maintaining ecosystem stability. The structure of the belowground microbiome is largely determined by individual plants, but it is not clear how far their influence extends and, conversely, what the influence of other plants growing nearby is.

**Results:**

To determine the extent to which a focal host plant influences its soil and root microbiome when growing in a diverse community, we sampled the belowground bacterial and fungal communities of three plant species across a primary successional grassland sequence. The magnitude of the host effect on its belowground microbiome varied among microbial groups, soil and root habitats, and successional stages characterized by different levels of diversity of plant neighbours. Soil microbial communities were most strongly structured by sampling site and showed significant spatial patterns that were partially driven by soil chemistry. The influence of focal plant on soil microbiome was low but tended to increase with succession and increasing plant diversity. In contrast, root communities, particularly bacterial, were strongly structured by the focal plant species. Importantly, we also detected a significant effect of neighbouring plant community composition on bacteria and fungi associating with roots of the focal plants. The host influence on root microbiome varied across the successional grassland sequence and was highest in the most diverse site.

**Conclusions:**

Our results show that in a species rich natural grassland, focal plant influence on the belowground microbiome depends on environmental context and is modulated by surrounding plant community. The influence of plant neighbours is particularly pronounced in root communities which may have multiple consequences for plant community productivity and stability, stressing the importance of plant diversity for ecosystem functioning.

**Supplementary Information:**

The online version contains supplementary material available at 10.1186/s40793-024-00550-z.

## Introduction

Fungi and bacteria in soil share microhabitats and resources and their interactions are fundamental to ecosystem functioning – they are key drivers of biogeochemical cycles and influence the health status and performance of plants [1]. Plants, in turn, substantially influence microbiome composition both associated with their roots and within their rhizospheres. However, although both soil fungi and bacteria rapidly react to the establishment of vegetation [2, 3], fungi seem to be more sensitive to vegetation composition turnover than bacteria [2, 4, 5].

Individual plants impact the soil habitat in species-specific manner through several pathways, including differences in root architecture and surface properties [6, 7], litter characteristics [8], and exudation patterns [9–11]. Indeed, plants have been repeatedly shown to leave species-specific imprints in the structure of soil microbial communities [12–16]. However, most previous studies were conducted on plant monocultures seldom encountered in natural ecosystems and their ecological validity might thus be limited [but see e.g. 17–19]. Plant species effect on soil microbiome is in large part mediated by root exudates [e.g. 20–22]. Their concentration is highest at the rhizoplane and decreases rapidly with distance from the root surface at a millimetre scale [23], limiting the range of plant influence to the immediate vicinity of their roots. In diverse, densely vegetated grasslands, however, roots of different plants touch and intertwine and can thus influence each other´s rhizosphere microbial communities directly, via microbial spillover from neighbouring roots, but also indirectly, by changing each other´s exudation patterns [24]. Together, these findings suggest that plants in diverse grassland mask each other´s effects on soil microbial communities which might lead to a homogenization of soil microbiota [25, 26].

Soil microbiome is the main source of fungi and bacteria colonizing plant roots, even though endophytes can be transmitted to roots also vertically from aboveground plant tissues [27–29]. The root is a particular environment consisting of two separate niches – rhizoplane and endosphere, with rhizoplane acting as a gate to the root interior [30]. Individual plants actively select their root-associated microbes and although some core bacterial and fungal taxa may be shared across groups of angiosperm plants [16, 31, 32], different plant species can typically be distinguished by the specific composition of their root microbiomes [e.g. 31, 33, 34].

Bacteria are small, mostly unicellular organisms with limited motility in the soil environment and thus constrained by their immediate surroundings [35]. As a result, plant host effect is likely to be particularly strong for root-associated bacteria which are under lower influence of external factors than rhizosphere bacteria. In contrast, root-associated fungi extend from the host root systems into the soil via hyphae and may even interconnect plants into a common mycelial network [36]. This implies that root-associated fungal communities are influenced not only by their host, but also by soil and neighbouring plants, as confirmed by recent studies [37, 38], and hints at a dilution of the host effect in the roots of plants growing in diverse plant communities, similar to that seen in the soil.

This study was designed to explore plant influence on the grassland microbiome in a realistic setting where plant individuals of different species live in a close proximity with other species of plants. We took advantage of a unique opportunity to study a grassland undergoing primary succession after deposition of soil recovered during limestone quarry extension where the studied focal plant species grew in the conditions of low to high diversity of surrounding plants. Bacterial and fungal components of the microbiome were analysed in the roots and soil of three focal plant species. We hypothesised that the influence of focal plant species (i) would be more prominent and constant in root than soil microbiome, (ii) that in soil, focal plant effect would be stronger for fungi compared to bacteria since major part of soil fungal communities consists of root-associated taxa extending into soil; in roots, plants should more influence bacteria that are restricted to the environment under root influence. Finally, we predicted that (iii) the focal plant effect in both roots and soil would decrease with increasing plant community diversity around the focal plant along a primary successional gradient.

## Materials and methods

### Sampling site

The study was carried out in a partially abandoned limestone quarry in the Czech Karst Protected Landscape Area in the Central Bohemia, Czech Republic (49° 57’ 46’’ N, 14° 10’ E). The study area is characterized by relatively warm climate and mild winters (mean annual temperatures 8–9 °C, mean annual precipitation 530 mm). The quarry´s abandoned parts have been progressively filled with clayey spoil since 2009 and left to spontaneous development. The oldest part of the landfill (ca. 150 × 100 m) represents an early successional habitat and is directly adjacent to a species-rich calcareous grassland, a late successional habitat, which serves as a source community for plant species colonizing the landfill [13]. The youngest part of the landfill that represents an initial stage of succession was deposited in 2014; in April 2015, it was sown with a mixture of grassland plant species to accelerate the establishment of the target grassland community that would have been seed dispersal-limited otherwise. The study system thus consisted of three grassland sites in different stages of their successional development (initial, early, late). The late successional dry grassland vegetation represented a dense community dominated by grasses (*Festuca rupicola*, *Arrhenatherum elatius*, *Poa pratensis*) and occurrence of numerous forb species and an occasional tree, with the total plant diversity 26 species per square meter (Červenková & Klinerová, unpublished data). Vegetation on the early successional grassland was also grass dominated, but less dense, consisted of both grassland and ruderal species with lower total diversity (12 species per square meter, Kuťáková, unpublished data). Vegetation on the initial site was rather patchy with high proportion of unvegetated soil and scarce occurrence of ruderal species and the sown dry grassland species (diversity 11 per square meter; Kuťáková, unpublished data). Though the species diversity on the initial site was very similar to the early successional site, the character of the vegetation is completely different as the initial site lacked dominance by grasses. These three grasslands thus form a unique study system for exploring factors influencing plant-microbiota interactions as they: (i) share the same plant species pool; (ii) are exposed to the same local climate; (iii) represent a natural gradient of vegetation complexity (richness/density). With the focal grassland species present at all sites, we could study species-specific root-associated microbiota across plant community diversity gradient.

**Fig. 1:**
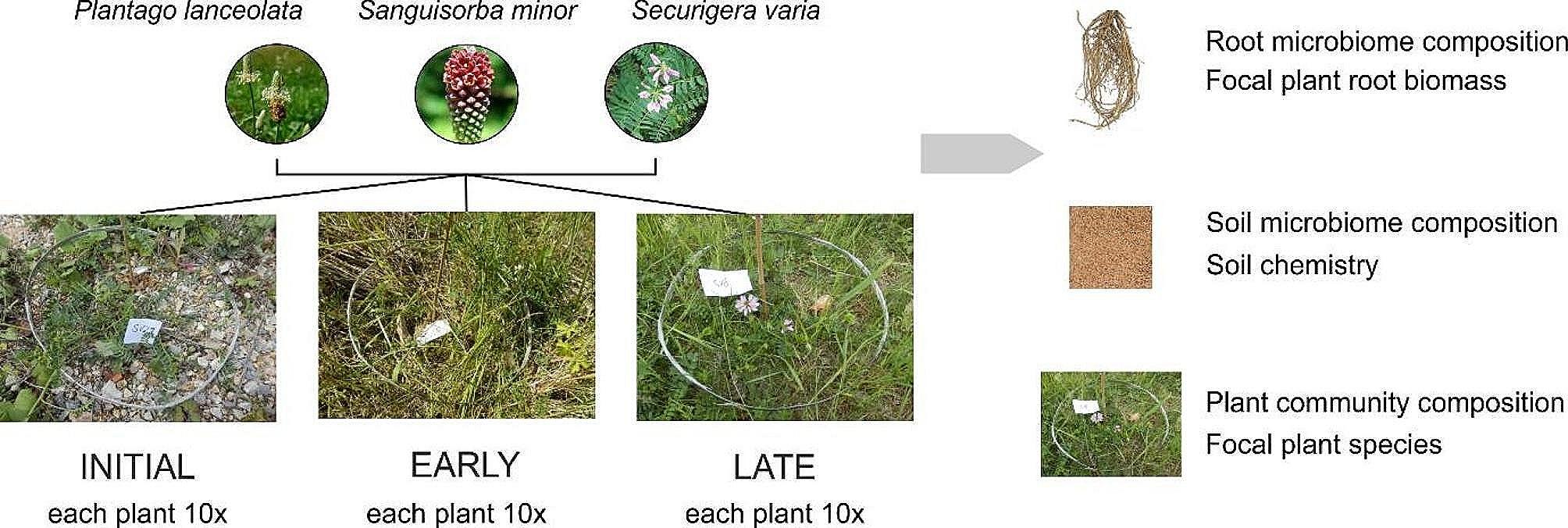
Sampling scheme

The sampling took place in June 2016. We selected three perennial plant species, *Plantago lanceolata* L. (*Plantaginaceae*), *Sanguisorba minor* Scop. (*Rosaceae*), and *Securigera varia* (L.) Lassen (Fabaceae), occurring on all three sites. We sampled 10 individuals per plant species per site (90 in total). Around each focal plant, we established a circular plot of 40 cm in diameter such that the selected plant was in its centre (Fig. 1).

Within each plot, we identified all vascular plant species and estimated their ground cover, the nomenclature followed Kubát et al. [39]. Next, we collected two soil cores (diameter 25 mm, depth 5 cm), one opposite the other, at the distance of ca. 10 cm from the focal plant to represent bulk soil under the influence of the focal plant. We acknowledge that below-ground biomass may not be uniformly distributed around the position of the plant, and that our sampling might have underestimated spatial heterogeneity. Finally, we carefully harvested the central plant, including the root system. All samples were transferred to the laboratory for processing. The two soil cores from each plot were pooled, litter and roots were removed, and the soil was sieved through a 5-mm sieve. All necessary measures were taken to prevent cross-contamination of soil samples. For each plant, we separated the aboveground part. Root system of each plant was thoroughly washed under tap water to represent a root sample. All the samples were freeze-dried, weighed and stored at -40 °C for further analyses. The freeze-dried roots were milled using an Ultra Centrifugal Mill ZM 200 (Retsch, Germany), and the resulting fine powder was used for the subsequent analyses.

### Soil chemistry analyses

To characterize soil chemical properties, soil samples were analysed for pH (1:10 w/v soil-to-water ratio), available phosphorus (P_avail_), total nitrogen (N_tot_) and soil organic matter (SOM) content. N_tot_ (using Carlo Erba Instrument NC 2500) and active pH [40] were measured by the Analytical laboratory of the Institute of Botany, Czech Academy of Sciences, Průhonice, Czech Republic. Soil organic matter (SOM) content was determined by the loss on ignition method [41]. Briefly, 5 g of soil were dried at 105 °C until constant weight and subsequently heated for 3 H at 550 °C (used oven: LAC LE15/11, HELAGO-CZ Ltd.). SOM (%) was calculated as (dry weight - weight after heating)/dry weight. P_avail_ was assessed in samples shaken with water (1:10, w:v) for 18 h, filtered through 0.22-μm membrane filter and measured with the malachite green method [42].

### DNA extraction and amplicon sequencing

Fungal and bacterial communities in soil and root samples were characterized by sequencing the internal transcribed spacer (ITS2) region and the V4 region of the 16 S ribosomal RNA gene, respectively. DNA from each freeze-dried sample (350 mg of soil, 30 mg of roots) was extracted in duplicates using the method of Miller modified by [43]. DNA extracts were purified using Geneclean Turbo Kit (Biogenic) following the manufacturer´s instructions, duplicates were pooled and stored in -20ºC before further use. For the microbial community analysis, PCR amplification of the fungal ITS2 region from DNA was performed using barcoded primers fITS7 (5′-GTGARTCATCGAATCTTTG-3′) and ITS4 (5′-TCCTCCGCTTATTGATATGC-3′) [44]. The V4 region of bacterial 16 S rRNA was amplified using the barcoded primers 515 F (5′-GTGCCAGCMGCCGCGGTAA-3′) and 806R (5′-GGACTACHVGGGTWTCTAAT-3′) [45]. PCR was performed in triplicate for each sample, and the resulting amplicons were purified, pooled, and libraries prepared with the TruSeq DNA PCR-Free Kit (Illumina) were sequenced in house on the Illumina MiSeq (2 × 250-base reads).

### Bioinformatic analysis

The amplicon sequencing data pre-processing was done using the pipeline SEED 2.0.3 [46]. Briefly, paired-end reads were merged using fastq-join [47] and fungal ITS2 region was extracted using ITSx 1.0.9 [48]. ITS sequences were then clustered at 97% similarity into Operational Taxonomic Units (OTUs) using UPARSE implemented in USEARCH7 [49], detected chimeric sequences were deleted. Most abundant sequences were then used as representative sequences for OTU identification using BLAST algorithm. The most abundant sequence was determined for each cluster, and the closest hits at a genus or species level were identified using blastn against the Ribosomal Database Project [50] and UNITE [51]. Sequences identified as nonbacterial or nonfungal were discarded. Fungal guilds were determined using FungalTraits database [52]. Based on literature research, we determined potentially plant-interacting bacterial genera belonging into one of following groups: endophytes, plant pathogens, plant-polymer degraders, plant growth promoters, bacteria involved in N cycle (N_2_-fixers, nitrifiers, denitrifiers), and plant-associated genera (genera often found in plant rhizosphere). While acknowledging the potential unreliability of functional classifications at the genus level, we believe that, when interpreted cautiously, such classifications can provide potentially interesting information on bacterial community composition. Sequence data have been deposited in the SRA under accession number PRJNA866350.

Illumina Miseq Sequencing yielded 2 605 041 bacterial and 3 009 837 fungal sequences. Samples were randomly subsampled to 10 000 reads except for those with lower read counts; samples with less than 1000 sequences were omitted. The resulting bacterial data set consisted of 1 526 851 sequences from 89 soil and 88 root samples. These sequences clustered into 23 746 OTUs including 9 593 singletons. The fungal dataset contained 1 645 749 fungal sequences from 90 soil and 90 root samples. These sequences clustered into 24 975 OTUs including 14 400 singletons. For microbial alpha diversity calculations, bacterial and fungal datasets were further subsampled to 4000 sequences and samples containing lower number of sequences (7 fungal and 7 bacterial) were omitted. The rest of statistical analyses were performed on datasets containing only OTUs occurring in at least 10% of samples (3214 bacterial OTUs, 94% of sequences; 849 fungal OTUs, 79% of sequences).

### Statistical analyses

All statistical analyses were performed using R software v. 4.1.0 [53], package *vegan* [54]. The R-Code used in this article is available from authors upon request.

The response datasets consisted of fungal and bacterial OTU composition for soil and root habitats, the explanatory datasets contained soil chemical variables (soil organic matter, total soil N, available soil P, pH), site characteristics (site identity, longitude, latitude, plant species richness, plant cover), and aboveground plant community composition. Bacterial and fungal species composition matrices were Hellinger-transformed prior to statistical analyses. Soil chemical variables were log-transformed to normalize distribution when necessary. Plant community data were square root transformed, summarized using principal component analysis (PCA) and reduced to the first five axes explaining 50.91% of variability (selected based on the broken-stick model) [55].

We measured bacterial and fungal species richness and beta diversity in soil and roots of different host species within sites of different successional age to characterise bacterial and fungal community dynamics during ecosystem complexity development. The beta diversity was calculated as Hellinger distance among samples. We used PCA to visualise the compositional differences among samples and projected the soil chemical variables onto the PCA ordination with the envfit function. This function calculates multiple regression of environmental variables with ordination axes and fits them as vectors onto the ordination diagram. The significance of the fit is tested via a randomised permutation procedure (999 permutations),

The effects of habitat (soil, root), site (initial, early successional, late successional) and focal plant identity on root and soil microbiomes were tested with PERMANOVA using the function adonis2. We reasoned that the site effect is, at least in part, mediated by soil chemistry and vegetation (i.e., plant community composition without the host) and has a strong spatial component. To examine the relative importance of these site attributes in structuring soil and root microbial communities, we performed the variation partitioning analysis [56] using the function varpart. The spatial predictors were derived from the GPS coordinates of individual samples using the principal coordinates of neighbourhood matrix (PCNM) method [57]. To select the most relevant predictors of microbial data variation, independent double-stop forward selections (*adespatial*) [58] of soil chemical variables, plant PC axes and PCNM vectors were carried out prior to the variation partitioning.

## Results

### Soil and vegetation characteristics

**Fig. 2:**
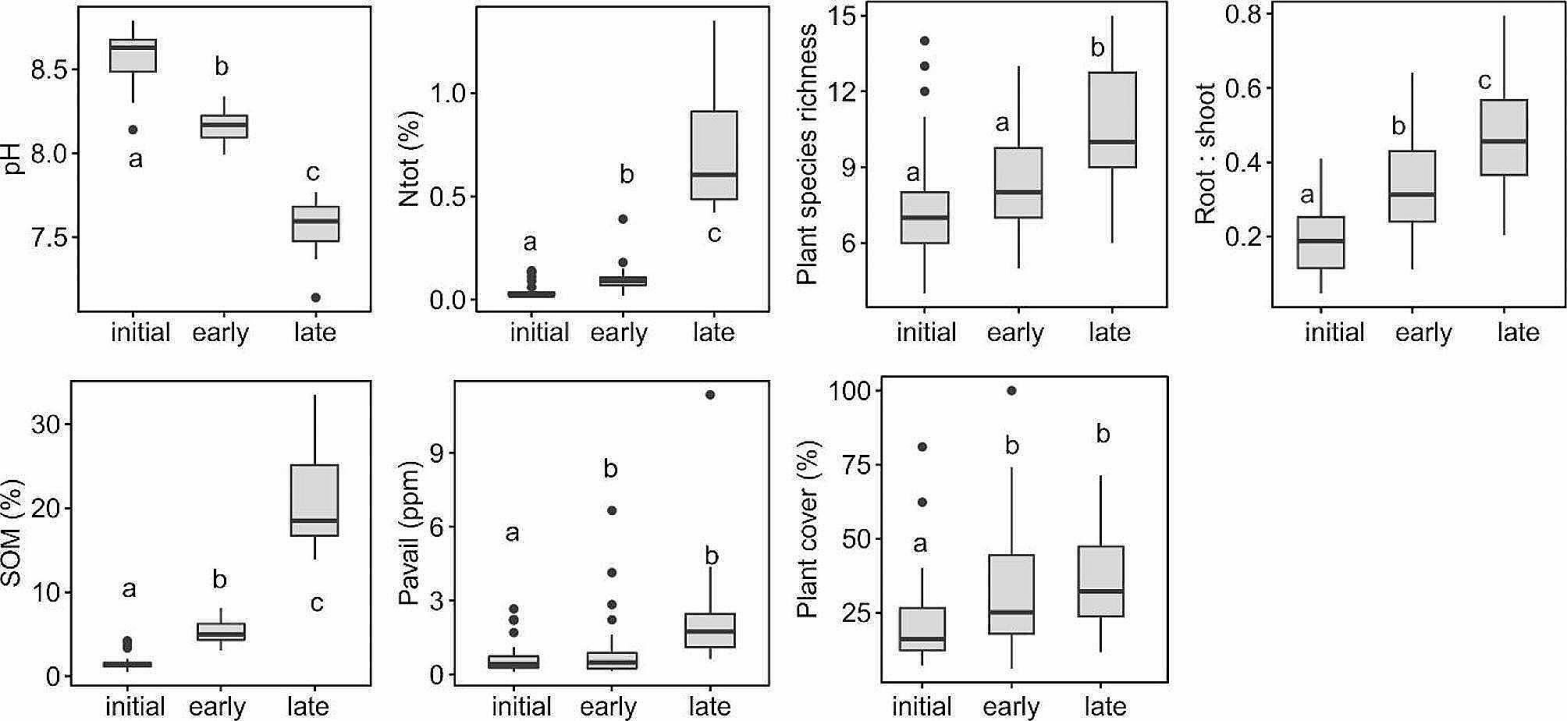
Soil chemical properties and plant community characteristics across a successional grassland sequence. Different letters in boxplots indicate significant differences between sites (*P* < 0.05), the differences were evaluated using ANOVA, followed by Tukey´s test of multiple comparisons

The three sites differed considerably in their soil chemical properties and vegetation. There was a clear gradient of pH, N_tot_, SOM and P_available_, with nutrient content increasing with site age and pH following the opposite direction (Fig. 2).

All soil chemical variables were substantially correlated (Fig. S2). The composition of plant communities surrounding individual focal plants followed the patterns observed on the three sites with species richness and plant cover increasing along the successional gradient. The root to shoot ratio of individual plants significantly increased with the ecosystem development (Fig. 2).

### Microbial community richness and composition

Bacterial and fungal species richness were significantly higher in soil than in roots across all sites (Table 1).

**Table 1:** Diversity of soil and root microbiomes. The results represent means and standard errors

		Bacteria			Fungi	
		Soil (*n* = 89)	Roots (*n* = 84)		Soil (*n* = 87)	Roots (*n* = 86)
Observed OTU richness		1102 ± 12 a	681 ± 17 b		410 ± 12 a	195 ± 5 b
Evenness		0.86 ± 0.00 a	0.78 ± 0.01 b		0.68 ± 0.01 a	0.55 ± 0.01 b
Chao-1		2235 ± 34 a	1344 ± 37 b		889 ± 32 a	427 ± 16 b

Means denoted by a different letter indicate significant differences between habitats (*P* < 0.05, Student´s t-test)

Soil bacterial richness tended to increase with pH and decrease with nutrient content while soil fungal richness followed an opposite pattern with fewer significant correlations that were also weaker. Root bacterial richness tended to decrease with pH and increase with soil nutrient content, plant species richness, plant cover and root to shoot ratio of focal plants. The species richness of root fungi did not show significant correlation with any variable (Fig. S3).

Bacterial and fungal communities differed in taxonomic composition between soil and root habitats on all sites (Fig. S4). The difference was primarily due to changes in relative abundance, not the presence/absence of particular microbial taxa, as the majority of both bacterial and fungal OTUs were shared between soil and roots and those OTUs that were unique to soil or roots were mostly rare, low-abundance OTUs constituting only a minor fraction of all reads (less than 10%, Fig. S5). The habitat was the strongest determinant of the bacterial community composition (PERMANOVA R^2^adj = 0.19, *P* < 0.001), followed by the site (R^2^adj = 0.16, *P* < 0.001), and focal plant identity (R^2^adj = 0.04, *P* < 0.001). The composition of fungal community was most strongly determined by the site (R^2^adj = 0.13, *P* < 0.001), and less by the habitat (R^2^adj = 0.05, *P* < 0.001), and the focal plant (R^2^adj = 0.02, *P* < 0.001; Fig. S4).

**Fig. 3:**
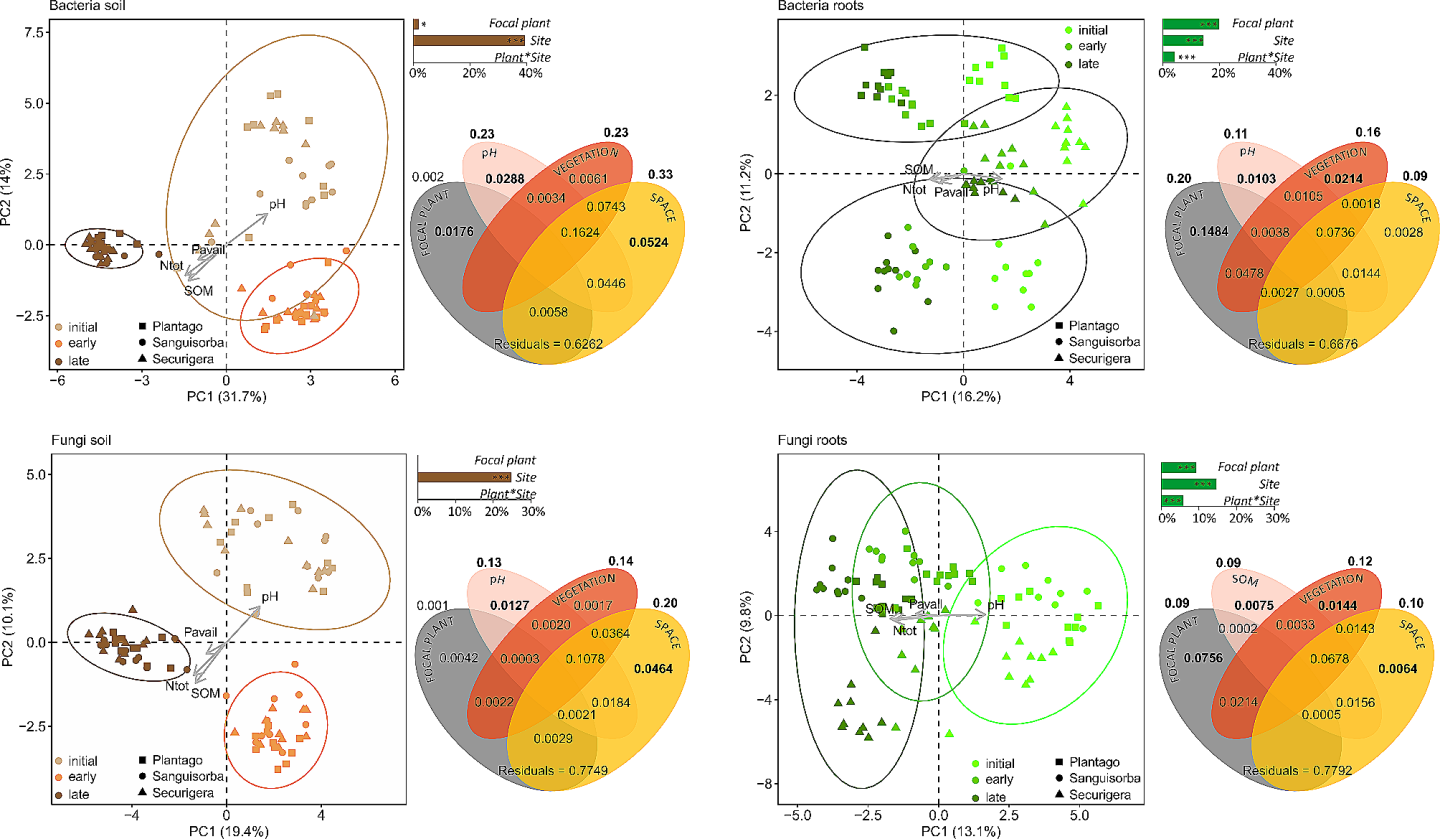
Composition of microbiomes in the roots and adjacent soil of *Plantago lanceolata, Sanguisorba minor*, and *Securigera varia* across a successional grassland sequence. Principal component analysis (PCA) ordination was calculated on Hellinger-transformed OTU abundances, environmental variables significantly correlated (*P* < 0.05) with ordination axes were added as vectors using envfit. Bar charts represent PERMANOVA outputs showing the amount of variation (adjusted R^2^) in the microbiota structure explained by the successional site, focal plant species, and their interaction, Venn diagrams depict the results of variance partitioning analysis. For each testable fraction, an adjusted R^2^ is given, and significance (*P* < 0.05) is indicated in bold. The values within the diagrams indicate the amount of variation explained purely by the given fraction, the values above the diagrams indicate the total variance explained by the given fraction, including the effect shared by the fraction and other variables

Within the habitats, the site was the best predictor of soil bacterial and fungal community composition and also of the root fungal community, whereas root bacterial community composition was most affected by focal plant identity (PERMANOVA results, Fig. 3).

Variation partitioning explained 22–37% of the variation in microbiome composition, depending on microbial group and habitat. Space and soil pH were the strongest drivers of both soil communities. Soil bacterial community was also significantly shaped by focal plant identity, while soil fungal community was not. Shared effects of pH and space explained more variation than pH alone, reflecting a spatial gradient of pH. Spatial structure and interrelationship with soil pH were also evident in the case of neighbouring plant community composition, whose influence manifested only through shared effects. The variation in root bacterial communities responded to all variables except space, while the root fungal community was significantly shaped by all variables. In both bacteria and fungi, the effect of neighbouring plant community on root microbiomes of the focal plant was significant. As with soil communities, a substantial fraction of variation in root communities was explained by the joint effects of space, soil chemistry and neighbouring plants (Fig. 3).

**Fig. 4:**
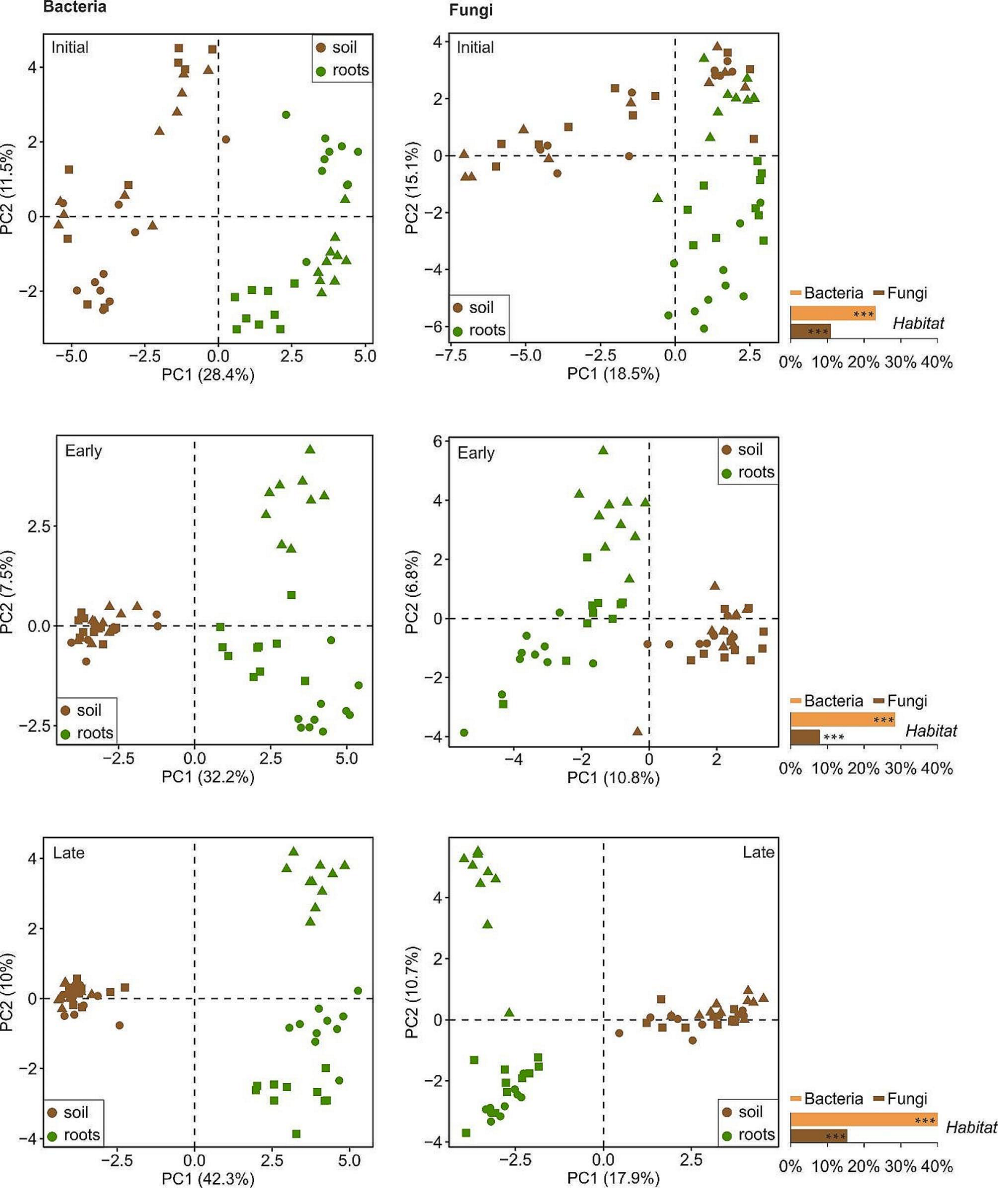
Bulk soil and root microbial communities of ■ *Plantago lanceolata*, ● *Sanguisorba minor*, and ▲ *Securigera varia* on initial, early and late successional sites. Principal component analysis (PCA) ordination was calculated on Hellinger-transformed OTU abundances. Bar charts represent PERMANOVA outputs showing the percent variation (adjusted R^2^) in the microbiota structure explained by the habitat (roots vs. soil)

To assess how the strength of individual factors changes with increasing ecosystem complexity across grassland succession, we tested their effects within individual sites using PERMANOVA and variation partitioning analysis. In the case of bacteria, the influence of habitat increased along the successional sequence, whereas fungal communities followed a slightly different pattern – the effect of habitat was lowest on the early successional site and higher in both initial and late successional sites (Fig. 4).

**Fig. 5:**
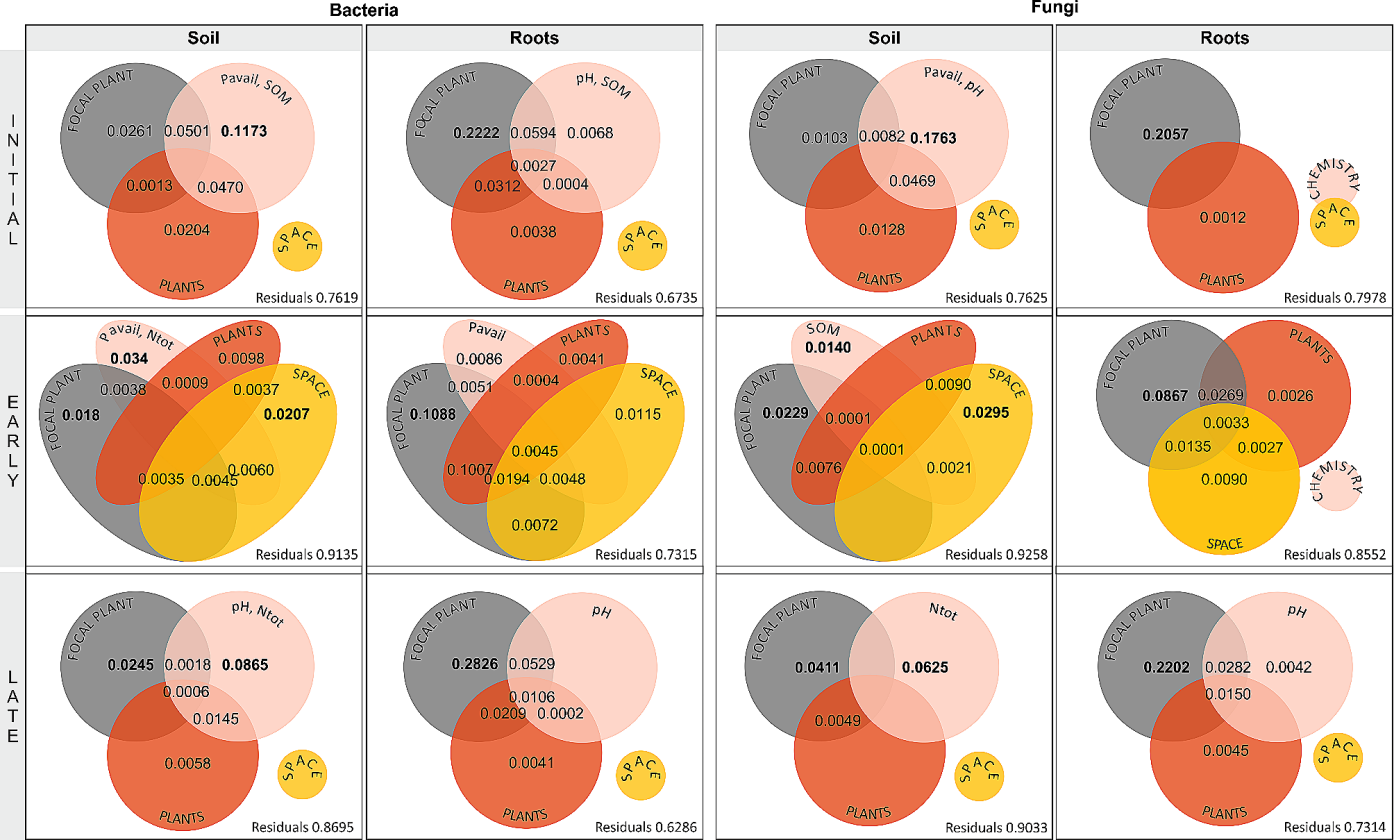
Variation in bulk soil and root-associated microbial communities within individual sites explained by focal plant species identity, soil chemistry, surrounding plant community composition (without focal plant), and space, and their joint effects. For each testable fraction, an adjusted R^2^ is given, and significant values (*P* < 0.05) are indicated in bold. Small empty circle indicates that no PCNM vector or chemical variable was significant for the community composition

The only significant driver of root-associated communities on all sites was focal plant identity whose influence was highest on the late successional site, slightly lower on the initial site and substantially lower on the early successional site. Soil bacterial and fungal communities were significantly affected by soil chemistry on all sites; its influence was highest in the initial soil and decreased through late successional to early successional site. The influence of focal plant on soil communities was insignificant on the initial site but increased along the successional gradient for both communities. Spatial structuring was detectable only in the early successional communities (Fig. 5).

## Discussion

In general, we were able to explain more variation in the composition of bacterial component of the microbiomes compared to the fungal one which is in agreement with a higher level of stochasticity and spatial variation in the composition of fungal communities [59–61]. Both bacterial and fungal communities showed significant level of habitat specificity across sites and hosts, however, this was much more evident in bacterial communities. When the individual habitats were analysed separately, the site was the dominant factor determining soil bacterial and fungal communities, as well as root fungal communities, but not root bacterial communities, which were primarily shaped by the focal plants.

### Soil microbial communities

Soil microbial communities across sites were significantly spatially structured. The spatial structure in microbial communities was partly driven by the spatial gradients in soil pH, a known driver of soil microbial communities [e.g. 62–64], and plant community composition, but a substantial part of it was due solely to spatial variables which might point to dispersal limitation of soil microbes [65].

Within individual sites, the effect of space has disappeared in all but the early successional site which is probably due to the fact that the early successional part of the grassland had the largest area and the average distance between early successional samples was significantly higher than between initial or late successional samples (Fig S1). Soil bacterial and fungal communities in the initial and late sites were relatively strongly shaped by soil chemistry and plant community composition. These results corroborate previous studies describing a high level of fine-scale heterogeneity of soil microbial communities [66, 67] and hint at underlying microgradients of environmental variables despite a seeming homogeneity within individual sites. In the early successional site, soil, plant and space variables explained only a small portion of the variation in the composition of soil communities. Compared to the initial site, the conditions in the early successional site are more benign which implies weaker environmental filtering of soil microbial communities [68], and at the same time, the competition is probably less fierce than in the late successional site [69]. Together, such conditions might lead to a lower selection pressure and greater stochasticity of soil microbial community assembly in the early successional site, which is further supported by the relatively higher diversity of early successional microbial communities.

Contrary to our hypothesis, the influence of focal plants on soil communities was rather weak for both bacterial and fungal communities but strengthened with succession. Plants colonising barren substrates bring about a surge of easily degradable nutrients in the form of root exudates and litter [70] and serve as hosts to microbial pathogens. Accordingly, the initial soil was significantly richer in putative plant pathogens and copiotrophic molds than the early and late successional soils which in turn harboured significantly more arbuscular mycorrhiza and plant associated bacteria, including plant growth promoters. Furthermore, initial and early soil were also richer in some putatively copiotrophic bacterial phyla/classes (Betaproteobacteria, Bacteroidetes) whereas grassland was rich in oligotrophic Planctomycetes and Verrucomicrobia (Fig. S4). This might suggest that plant colonisation of barren substrate initially non-selectively boosts the growth of microbial opportunists and copiotrophs, but later, as the ecosystem develops, soil microbial communities become more plant-oriented and positive plant-microbe interactions become more common [71].

### Root microbial communities

In line with our hypotheses the effect of the focal plant on the root microbiome was much stronger than on the soil microbiome. Moreover, whereas bacterial root communities across sites were shaped primarily by focal plants, fungal communities were largely determined by site and focal plant was less important. In contrast to our expectations, the focal plant effect did not wane with increasing vegetation/ecosystem complexity but was U-shaped: it was similarly strong on the late successional site and the initial site and relatively weak on the early successional site. The vegetation on the initial site was established by sowing one year before the sampling at which time the site was completely devoid of plants. At the time of sampling, the soil contained very little nutrients and plant-derived compounds thus likely represented major nutrient source for the soil and, even more so, root microbes. Furthermore, plant density was low; focal plants experienced little to no interference from neighbouring plants in shaping their associated microbiotas. Late successional soil, on the other hand, was relatively rich in nutrients and densely covered with species rich vegetation. Focal plants on the late successional site had the greatest root to shoot ratio, which suggests stronger belowground competition pressure [72], and thus interference, from neighbouring plants. However, the host species effect on microbial community structure was actually strongest on the late successional site and we could only observe the expected decrease of focal plant influence and simultaneous increase of the influence of plant neighbours in the early successional samples. This might suggest that the link between plant hosts and their root-associated microbes strengthen during ecosystem development. We did not analyse the total root biomass on the three sites, however, given that the plant cover and diversity, as well as the root to shoot ratio, increased with succession, it is likely that the amount and biomass of roots also increased. These roots might act as a reservoir of microbial colonists for next generations of plants [12] which underlies the observed consolidation of host effect.

Importantly, our results confirm that in addition to the effect of the host plant, the microbiome of living roots is affected by the composition of the plant community around the focal plant. While for fungi, this can be due to the direct contact between roots of plants through growing hyphae, in bacteria, the possible mechanism may be the direct contact of roots. Still, this observation indicates that within an ecosystem, plant species do not live in isolation and their microbiomes reflect the complexity of the grassland environment.

## Conclusion

In this work, we investigated the extent to which a host plant is able to influence its soil and root microbiome when grown in a diverse community, and how the strength of host influence varies with increasing ecosystem complexity. The magnitude of the host effect on soil and root microbes varied among microbial groups, habitats, and successional sites. The strength of association of the microbiome with its habitat, as well as host effect on soil microbial communities, increased with succession in both bacteria and fungi. In contrast, host effect on root communities was strongest at the initial and late stages of succession, i.e. under relatively stressful conditions of low resources and high competition, respectively, and moderate at the early stage, when the conditions were more favourable. Importantly, we show that root communities of bacteria and fungi bear the imprint of plants growing in close proximity of their host which expands our understanding of the link between plant diversity and belowground microbiome.

### Electronic supplementary material

Below is the link to the electronic supplementary material.

**Supplementary Material 1:** Supplementary tables - Vegetation data; Bacterial OTU table; Fungal OTU table; Sample metadata

**Supplementary Material 2: Figure S1.** PCA biplot showing the structure of the plant community across a successional grassland sequence and a satellite map showing the distribution of the sampling points. PCA was calculated on sqrt-transformed plant cover data, the 10 dominant plant species depicted in the biplot were selected based on the highest cos2 value. **Figure S2.** Pairwise scatter plot matrix (lower panel), frequency distribution histogram (main diagonal), and Spearman correlation coefficients (upper panel) of soil chemical variables across a successional grassland sequence * p < 0.05; ** p < 0.01; *** p < 0.001. **Figure S3.** Bacterial and fungal diversity across sites and its correlation with pH, plant species richness and plant cover. Different letters in the boxplots denote statistically significant differences at P < 0.05 determined by ANOVA. Red lines in the scatter plots represent linear regression models at P < 0.05 and 95% confidence intervals are visualized as grey shaded areas. **Figure S4.** Soil and root microbiome communities associated with *Plantago lanceolata*, *Sanguisorba minor*, and *Securigera varia* across a successional grassland sequence. A) Principal component analysis (PCA) ordination was calculated on Hellinger-transformed OTU abundances, environmental variables significantly correlated (P < 0.05) with ordination axes were added as vectors using envfit. Bar chart represents a PERMANOVA output showing the amount of variation (adjusted R^2^) in the microbiota structure explained by the habitat (soil/roots), successional site and focal plant species. B) Fungal and bacterial ecological guilds and bacterial phyla in **s**oil and roots across a successional grassland sequence. The values represent mean relative abundances (n = 30), different letters denote statistically significant difference in relative abundance at p < 0.05 (one-way ANOVA). Note that we did not test the differences among root samples. **Figure S5.** Distribution of bacterial (left) and fungal (right) OTUs between the studied habitats. Numbers in italics in the Venn diagrams indicate the percent contribution of the habitat specific or shared OTUs to all OTUs (*%OTU*) and the fraction of all reads they represent (*%Seq.*) Venn diagrams were created using sequence datasets where singletons and doubletons were omitted and read counts per sample were rarefied to the minimum sample depth

## Data Availability

Sequence data were deposited in the NCBI Short Read Archive under BioProject PRJNA866350. Processed data are available as Supporting information and the R-Code used in this article is available from authors upon request.
