# Supplementary material for: Plant effects on microbiome composition are constrained by environmental conditions in a successional grassland
Source: Environ Microbiome. 2024 Jan 24;19:8. doi: 10.1186/s40793-024-00550-z (PMC10809484; doi:10.1186/s40793-024-00550-z)
Supplement: Supplementary file 2 — Supplementary Material 2: Figure S1. PCA biplot showing the structure of the plant community across a successional grassland sequence and a satellite map showing the distribution of the sampling points. PCA was calculated on sqrt-transformed plant cover data, the 10 dominant plant species depicted in the biplot were selected based on the highest cos2 value. Figure S2. Pairwise scatter plot matrix (lower panel), frequency distribution histogram (main diagonal), and Spearman correlation coefficients (upper panel) of soil chemical variables across a successional grassland sequence * p < 0.05; ** p < 0.01; *** p < 0.001. Figure S3. Bacterial and fungal diversity across sites and its correlation with pH, plant species richness and plant cover. Different letters in the boxplots denote statistically significant differences at P < 0.05 determined by ANOVA. Red lines in the scatter plots represent linear regression models at P < 0.05 and 95% confidence intervals are visualized as grey shaded areas. Figure S4. Soil and root microbiome communities associated with Plantago lanceolata, Sanguisorba minor, and Securigera varia across a successional grassland sequence. A) Principal component analysis (PCA) ordination was calculated on Hellinger-transformed OTU abundances, environmental variables significantly correlated (P < 0.05) with ordination axes were added as vectors using envfit. Bar chart represents a PERMANOVA output showing the amount of variation (adjusted R2) in the microbiota structure explained by the habitat (soil/roots), successional site and focal plant species. B) Fungal and bacterial ecological guilds and bacterial phyla in soil and roots across a successional grassland sequence. The values represent mean relative abundances (n = 30), different letters denote statistically significant difference in relative abundance at p < 0.05 (one-way ANOVA). Note that we did not test the differences among root samples. Figure S5. Distribution of bacterial (left) and fungal (r [file 40793_2024_550_MOESM2_ESM.pdf]

SUPPLEMENTARY MATERIAL

Plant effects on microbiome composition are constrained by environmental conditions in a successional grassland

Lenka Mészárošová, Eliška Kuťáková, Petr Kohout, Zuzana Münzbergová, Petr Baldrian

Supplemental Figures pages 2-6

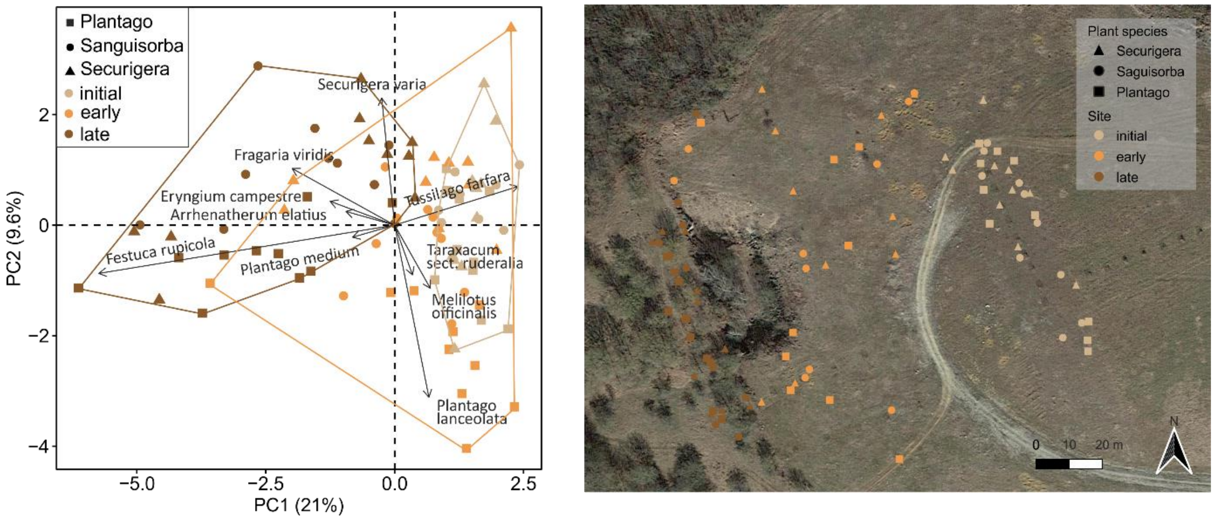

Figure S1. PCA biplot showing the structure of the plant community across a successional grassland sequence and a satellite map showing the distribution of the sampling points. PCA was calculated on sqrt-transformed plant cover data, the 10 dominant plant species depicted in the biplot were selected based on the highest cos2 value.

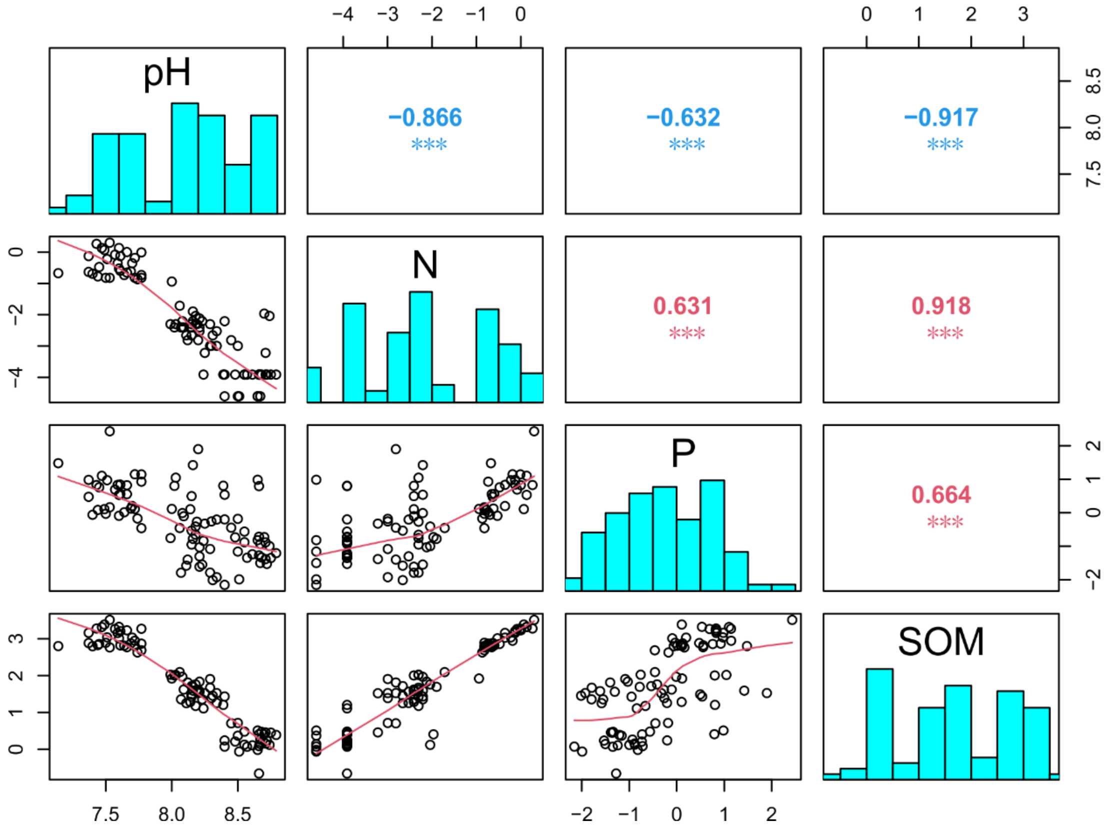

Figure S2. Pairwise scatter plot matrix (lower panel), frequency distribution histogram (main diagonal), and Spearman correlation coefficients (upper panel) of soil chemical variables across a successional grassland sequence * $p < 0.05$; ** $p < 0.01$; *** $p < 0.001$

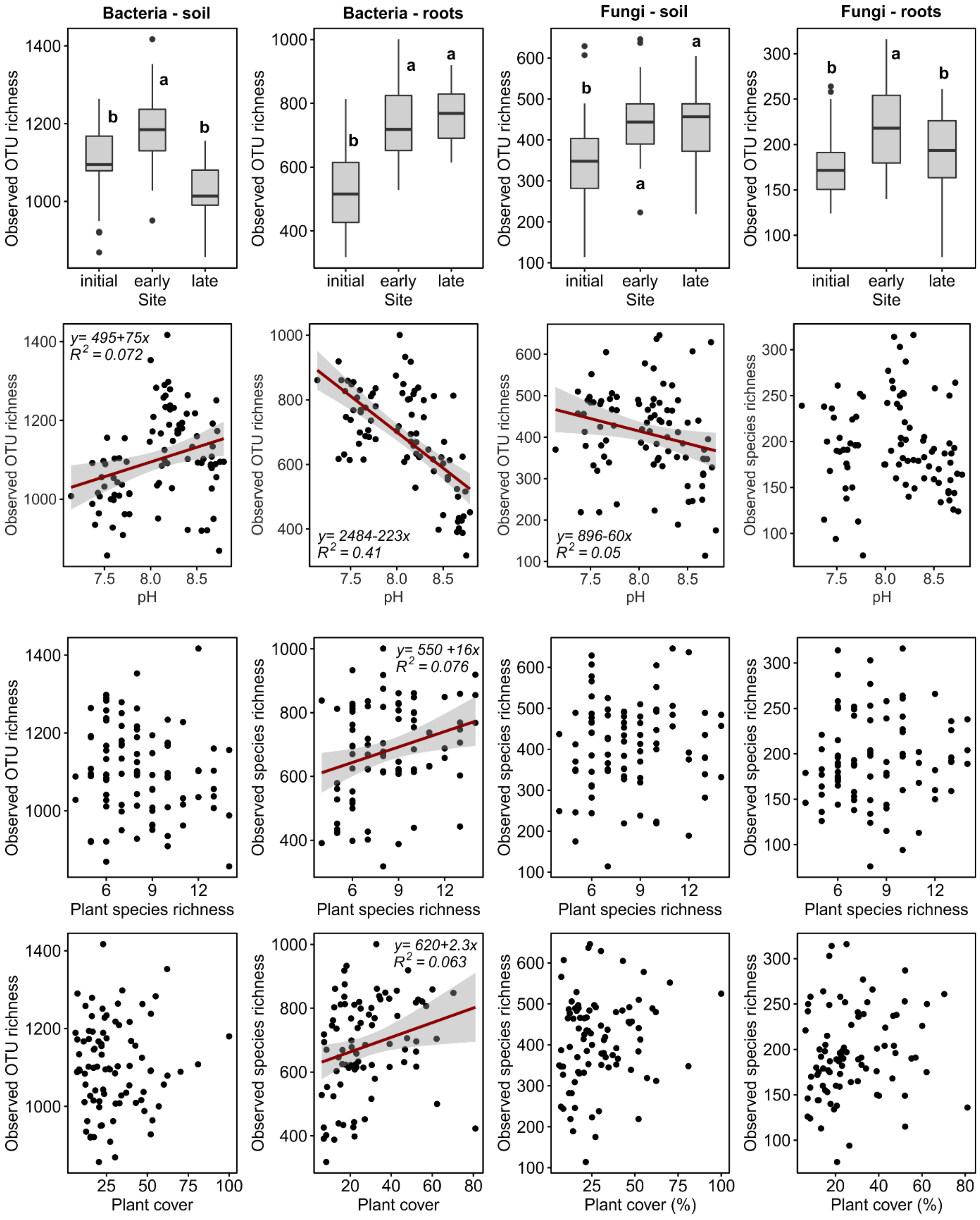

Figure S3. Bacterial and fungal diversity across sites and its correlation with pH, plant species richness and plant cover. Different letters in the boxplots denote statistically significant differences at $P < 0.05$ determined by ANOVA. Red lines in the scatter plots represent linear regression models at $P < 0.05$ and 95% confidence intervals are visualized as grey shaded areas.

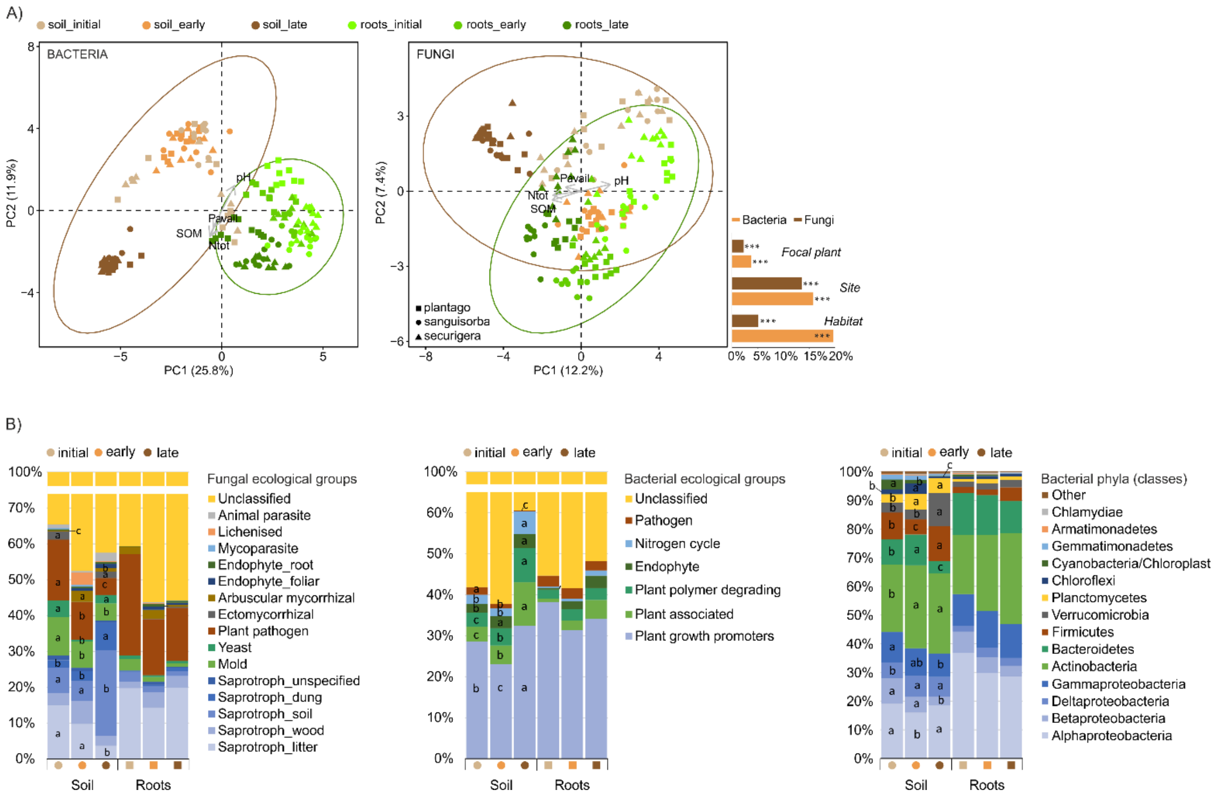

Figure S4. Soil and root microbiome communities associated with *Plantago lanceolata*, *Sanguisorba minor*, and *Securigera varia* across a successional grassland sequence.

A) Principal component analysis (PCA) ordination was calculated on Hellinger-transformed OTU abundances, environmental variables significantly correlated (P < 0.05) with ordination axes were added as vectors using envfit. Bar chart represents a PERMANOVA output showing the amount of variation (adjusted R^2) in the microbiota structure explained by the habitat (soil/roots), successional site and focal plant species. **B)** Fungal and bacterial ecological guilds and bacterial phyla in **s**oil and roots across a successional grassland sequence. The values represent mean relative abundances (n = 30), different letters denote statistically significant difference in relative abundance at p < 0.05 (one-way ANOVA). Note that we did not test the differences among root samples.

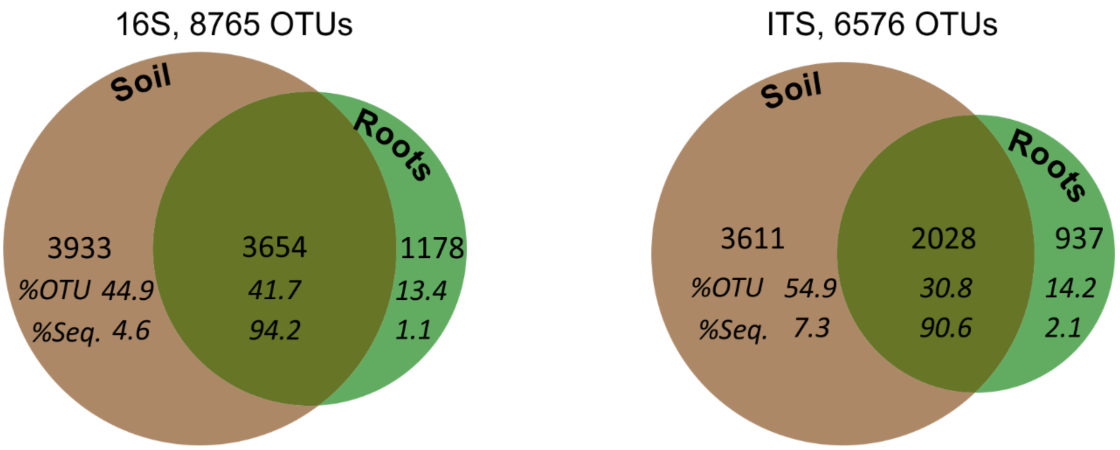

Figure S5. Distribution of bacterial (left) and fungal (right) OTUs between the studied habitats. Numbers in italics in the Venn diagrams indicate the percent contribution of the habitat specific or shared OTUs to all OTUs (*%OTU*) and the fraction of all reads they represent (*%Seq.*) Venn diagrams were created using sequence datasets where singletons and doubletons were omitted and read counts per sample were rarefied to the minimum sample depth.
